# Real-World Use and Predictors of Response to Disopyramide in Patients with Obstructive Hypertrophic Cardiomyopathy

**DOI:** 10.3390/jcm12072725

**Published:** 2023-04-06

**Authors:** Niccolò Maurizi, Chiara Chiriatti, Carlo Fumagalli, Mattia Targetti, Silvia Passantino, Panagiotis Antiochos, Ioannis Skalidis, Chiara Chiti, Giulia Biagioni, Alessia Tomberli, Sara Giovani, Raffaele Coppini, Franco Cecchi, Iacopo Olivotto

**Affiliations:** 1 Department of Clinical and Experimental Medicine, University of Florence, 50121 Florence, Italy; 2 Service of Cardiology, University Hospital of Lausanne, 1009 Lausanne, Switzerland; 3 Cardiomyopathy Unit, Careggi University Hospital, 50134 Florence, Italy; 4 Department NeuroFarBa, University of Florence, 50121 Florence, Italy; 5 Fondazione AICARM, 50100 Florence, Italy; 6 Service of Cardiology, Meyer Children’s Hospital IRCCS, 50139 Florence, Italy

**Keywords:** hypertrophic cardiomyopathy, disopyramide, obstructive HCM management

## Abstract

**Background:** Although disopyramide has been widely used to reduce left ventricular outflow obstruction (LVOTO) and to improve symptoms in patients with obstructive hypertrophic cardiomyopathy (oHCM), its use in real world as well as patient characteristics associated with a positive treatment response are still unclear. **Methods:** From 1980 to 2021, 1527 patients with HCM were evaluated and 372 (23%) had a LVOTO with active follow-up. The efficacy and safety of disopyramide were assessed systematically during 12 months (2-, 6-, and 12-month visits). Responders were patients with a final NYHA = I and a LVOTO < 30 mmHg; incomplete responders were those patients with NYHA > I and a LVOTO < 30 mmHg; and non-responders were symptomatic patients with no change in functional class NYHA and a LVOT gradient > 30 mmHg. **Results:** Two-hundred-fifty-four (66%) patients were in functional class NYHA I/II and 118 (34%) in NYHA III/IV. A total of 118/372 (32%, 55 ± 16 years) underwent disopyramide therapy. Twenty-eight (24%) patients responded to therapy, 39 (33%) were incomplete responders, and 51 (43%) did not respond. Responder were mainly patients in functional NYHA class I/II (24/28, 86%), whereas incomplete responders and non-responders were more often in functional NYHA class III/IV (50/54 (93%)). An independent predictor of response to disopyramide treatment was the presence of NYHA I/II at the initiation of therapy (HR 1.5 (95% CI 1.1–4.5), *p* = 0.03). No major life-threatening arrhythmic events or syncope occurred, despite 19 (16%) patients showing reduced QTc from baseline, 19 (16%) having no difference, while 80 (69%) patients had prolonged QTc interval. Thirty-one (26%) patients experienced side effects, in particular, 29 of the anticholinergic type. **Conclusions:** Disopyramide was underused in oHCM but effective in reducing LVOTO gradients and symptoms in slightly symptomatic patients with less severe disease phenotype with a safe pro-arrhythmic profile.

## 1. Introduction

Hypertrophic cardiomyopathy (HCM) is the most common genetic cardiomyopathy, characterized by heterogeneous phenotype and clinical course [1,2].

Among the earliest functional manifestations of disease are a hyperdynamic ventricular contraction and impaired relaxation. Dynamic left ventricular outflow tract (LVOT) obstruction due to systolic anterior motion (SAM) of the mitral valve and contact with the interventricular septum is a hallmark and one of the determinants of the clinical course in HCM [3]. In recent years, several drugs addressing promising therapeutic targets have been investigated in obstructive HCM, but have failed to demonstrate their efficacy or proved to be poorly tolerated [4]. Therefore, the treatment of symptomatic patients with obstructive HCM is still mainly based on negative inotropic drugs such as beta-blockers (BB) and/or non-dihydropyridine calcium-channel blockers (CCBs; primarily verapamil) [5]. In patients with resistant symptoms, disopyramide can be employed as a second-line therapy, owing to its strong negative inotropism, increase in systemic resistances, and ability to decrease early LV ejection flow acceleration [5].

Data concerning the safety and efficacy of this disopyramide in obstructive HCM are limited from a few studies [6,7,8,9] and still many open questions remain. As an example, patient characteristics associated with treatment response are unclear and incertitude is still present concerning in which disease stage disopyramide would be the most effective [9]. This is of critical relevance since its anti-cholinergic and very rare pro-arrhythmic side effects may limit its use [6,7]. Moreover, the recent development of disease-specific therapies represents not only a major opportunity for HCM patients but also a challenge for the clinician, since the appropriate and timely treatment selection for the correct patient’s sub-group is now a priority [10]. Lastly, safety concerns remain, partly due to its anticholinergic effects [8] and its blockage of the rapid delayed rectifier cardiac potassium current (I Kr), with a potential significant QT-prolonging effect [11]. Therefore, we evaluated the real-world use of disopyramide in a large cohort of obstructive HCM, by determining its safety and efficacy in reducing LVOT gradients and symptoms, as to identify possible patient responder sub-groups.

## 2. Methods

### 2.1. Study Population

We analyzed the clinical data and management data of 1527 HCM patients consecutively diagnosed at our center from 1980 to 2021. Of these, 372 (25%) had a left ventricular outflow tract obstruction (LVOTO) with active follow-up. Diagnosis was based on two-dimensional echocardiographic evidence of a hypertrophied, non-dilated LV (maximum wall thickness ≥ 15 mm, or the equivalent relative to body surface area in children), in the absence of another cardiac or systemic disease capable of producing the magnitude of hypertrophy evident. The peak LV outflow tract velocity was averaged from 3 to 5 cardiac cycles recorded at a sweep speed of 50 to 100 mm/s. Outflow obstruction was defined using Doppler echocardiography as a systolic anterior motion of the mitral valve and LV outflow tract (subaortic) velocity of >2.7 m/s at rest of >3.5 m/s with provocation, which is respectively comparable to an outflow gradient > 30 mm Hg at rest or >50 mm Hg with provocation. In all cases, particular consideration was given to distinguishing the Doppler signal of LVOTO from that of mitral regurgitation.

### 2.2. Patient Evaluation and Management

Patients with a diagnosis of HCM presenting with LVOT obstruction were evaluated, according to current ESC Guidelines [5] or up to date best practice, as follows:

1. Patients with a function class NYHA I and symptoms related to LVOT obstruction or NYHA II were treated with non-vasodilating beta-blockers titrated to maximum tolerated dose or, if contraindicated verapamil (starting dose 40 mg three times daily to maximum 480 mg daily);
2. Patients with a function class NYHA I and symptoms related to LVOT obstruction or NYHA II-III, after ineffective 6-month treatment with beta-blockers/verapamil, disopyramide was introduced up to a maximum tolerated dose (usually 400–500 mg/day). Exclusion criteria for disopyramide initiation were glaucoma, men with prostatism, patient with baseline QTc > 550 msec, and those with LVEF < 50%;
3. Patients with an LVOTO gradient ≥ 50 mm Hg, moderate-to-severe symptoms (New York Heart Association (NYHA) functional Class III–IV), and/or recurrent exertional syncope in spite of maximally tolerated negative inotropic therapy were proposed with an invasive management of LVOT gradient.

Disopyramide was initiated at the routine initial dose of 125 mg short-acting disopyramide two times daily. At the day of disopyramide initiation, an electrocardiogram (ECG) and echocardiogram are performed. During each clinical follow-up, patients underwent resting 12-lead ECG, resting echocardiography with provocation, and a questionnaire concerning possible side effects of disopyramide. A 2-, 6-, and 12-month follow-up was scheduled for each patient. From the 6-month visit, according to the clinical response, increasing the disopyramide dose or discontinuation of the drug was considered. Pyridostigmine, which has been demonstrated to attenuate the anticholinergic side effects of disopyramide, was also considered if patients developed such side effects. An intermediate clinical visit was performed at 6 months and the last visit including ECG, and echocardiography was performed at 12 months from therapy initiation. Special care was taken to monitor the side effects of the drug in patients with renal or hepatic impairment attributed to potential effects on drug clearance, in those with atrial fibrillation and atrial flutter because of the potential for disopyramide-induced augmentation of atrioventricular conduction and increased ventricular rate. The decision to initiate disopyramide therapy is taken by the treating physician as is the tailoring of the above-mentioned protocol to the individual patient.

### 2.3. Twelve-Lead Electrocardiogram Analysis

Each ECG was recorded on the visit day. PR, QRS, and QT intervals were measured manually by a single investigator experienced in ECG interpretation (C.C.). QT intervals were measured using the tangents method using lead II. If measurement in lead II was impossible because of technical limitations, leads V5 or V2 were used in this order. The correction for heart rate was performed using Fridericia’s formula. Although Bazett’s formula is the most commonly used, it is less accurate in relative tachycardia or bradycardia and therefore less reliable when comparing ECGs with different heart rates. Accordingly, Fridericia’s formula is recommended over Bazett’s formula for the evaluation of drug-induced QT prolongation (http://www.fda.gov/downloads/drugs/guidancecomplianceregulatoryinformation/guidances/ucm073153.pdf, accessed on 7 March 2023). Patients who were ventricularly paced were excluded from ECG analysis.

### 2.4. Definition of the Response to Disopyramide 

At the 12-month follow-up, patients were categorized based on the clinical and echocardiographic response to negative inotropic agents, specifically:

1. Responders: patients with a functional class NYHA = I and a LVOT gradient < 30 mmHg.
2. Incomplete responders: patients with a functional class NYHA > I and a LVOT gradient < 30 mmHg.
3. Non-responders: symptomatic patients with no change in functional class NYHA and a LVOT gradient > 30 mmHg.

### 2.5. Statistical Analysis

Continuous variables, reported as means with standard deviations or as medians with interquartile ranges for non-normal distributions, were compared between groups with the Student *t* tests or non-parametric tests, as appropriate. Categorical variables, reported as counts and percentages, were compared between groups with χ^2^ tests or Fisher exact tests when any expected cell count was less than 5.

Cox multivariable regression analysis (variable selection method with backward stepwise elimination) was performed including all candidate variables (*p* < 0.10 at univariate analysis). A 2-sided *p*-value less than 0.05 was considered statistically significant. All analyses were performed using SPSS Statistics for Macintosh version 25.0 (IBM).

## 3. Results

### 3.1. Clinical and Echocardiographic Profile of Patients with Obstructive HCM

**Figure 1:**
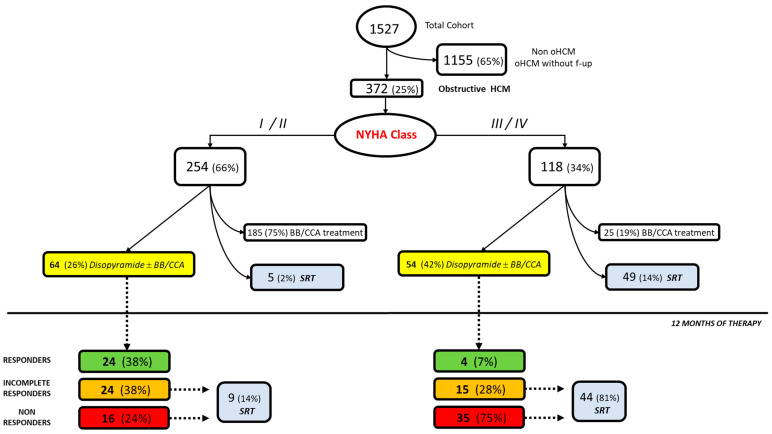
Clinical management of patients with obstructive hypertrophic cardiomyopathy. *Abbreviations: oHCM: obstructive hypertrophic cardiomyopathy; f-up: follow-up; BB: beta-blockers; CCA: calcium antagonists; SRT: septal reduction treatments.*

Of the 1527 patients diagnosed with HCM in our center from 1981 to 2021, 372 (25%) were diagnosed with obstructive HCM had an active follow-up. Of these, 254 patients (66%) were in functional class NYHA I/II and 118 (34%) in NYHA class III/IV (Table 1, Figure 1). The mean age at therapy initiation was 43 ± 19 years and 226 (61%) were males. Specifically, among patients in NYHA Class I/II, 185 (75%) were on BB/CCA treatment, 5 (2%) directly underwent septal reduction therapies (SRTs), whereas 64 (26%) were on disopyramide ± BB/CCA treatment (Figure 1). Highly symptomatic patients, in functional class NYHA III/IV, received negative inotropic drugs (25 (19%) on BB/CCA treatment), disopyramide ± BB/CCA (54 (42%)), and direct SRT (49 (14%; Figure 1). Therefore, a total of 118 patients (63 (53%) males, 55 ± 16 years) underwent therapy with disopyramide at the initial 250 mg dose in addition to beta-blocker/calcium channel blocker therapy. One hundred and eleven (94%) patients presented functional limitations (NYHA > I) at the beginning of therapy, and 32 (27%) presented at least an episode of atrial fibrillation. Systolic anterior motion of the mitral valve (SAM) was present in 95 (80%) patients, whereas the mean left atrial diameter was 45 ± 7 mm. A total of 76 (64%) presented a LVOT obstruction > 50 mmHg and mean left ventricular ejection fraction (LV EF) of 66 ± 7 % (Table 1).

Patients who underwent disopyramide therapy presented with, before treatment initiation, a first-degree atrioventricular block (AVB) in 10 (8%) cases, a bundle branch block (BBB) in 14 (11%), a QTc interval > 480 msec in 30 (24%), and a QTc > 500 msec in 14 (11%) (Table 2).

### 3.2. Efficacy and Criteria of Response to Disopyramide Therapy 

After 12 months of therapy, an improvement in the functional class occurred. Five (4%) patients in NYHA II class became asymptomatic; 21 (25%) patients improved their functional class from NYHA III to NYHA II. The symptomatic relief did not differ in patients who took a dose of 250 mg/day vs. higher doses (*p* = 0.79). The mean LVOTO gradient at rest post-therapy was not abolished but was significantly reduced (72 ± 36 mmHg vs. 49 ± 31 mmHg; *p* < 0.001). Specifically, 28 (24%) were responders to therapy, 39 (33%) were incomplete responders, and 51 (43%) did not respond to therapy. Among the latter, 53 (45%) underwent subsequent SRT (9/64 (14%) in the NYHA I/II and 44/54 (81%) in the NYHA III/IV group) (Figure 1). Responders were mainly patients in functional NYHA class I/II (24/28, 86%), whereas incomplete responders and non-responders were more often patients in functional NYHA class III/IV (50/54 (93%) (Table 1, Figure 1). Moreover, responders were younger (48 ± 9 mm vs. 51 ± 11 mm and 58 ± 12 mm for incomplete responders and non-responders, *p* for trend < 0.01), had a smaller left atrium (42 ± 5 mm vs. 44 ± 5 mm and 46 ± 5 mm for incomplete responders and non-responders, *p* for trend < 0.01), and less severe LVOT gradient (15 (53%) with LVOT > 50 mmHg vs. 29 (74%) and 32 (63%) for incomplete and non-responders, *p* for trend < 0.01). Non-responders, as compared to the two other groups, had also a lower LV EF (63 ± 8 vs. 66 ± 6 and 69 ± 8 for incomplete responders and responders, *p* for trend < 0.01) (Table 1).

Factors associated with response to disopyramide therapy were age (per 10 decrease) (HR 1.4 (95% CI 0.5–3.6), *p* = 0.03), left atrial diameter (per 2 mm decrease) (HR 2.1 (95% CI 0.9–7.8), *p* < 0.01), LV EF (HR 4.2 (95% CI 1.3–9.9), *p* < 0.01), and NYHA Class I/II at therapy initiation (HR 5.1 (95% CI 2.3–11.2), *p* < 0.01). The latter was the only multivariable predictor of response to disopyramide treatment (HR 1.5 (95% CI 1.1–4.5), *p* = 0.03) (Table 3).

### 3.3. Safety of Disopyramide Therapy

During the therapy, no major life-threatening arrhythmic events or syncope occurred.

Atrioventricular conduction was prolonged during treatment: the mean PR interval pre-treatment was 178 ± 22 msec in a total of 10 patients (8%) with AVB I vs. 183 ± 24 msec in 22 patients (17%) with AVB I after treatment, *p* < 0.01. No increase in intraventricular conduction was observed, and the median prolongation of the QTc interval was 21 [2; 32] msec (Table 2).

**Figure 2:**
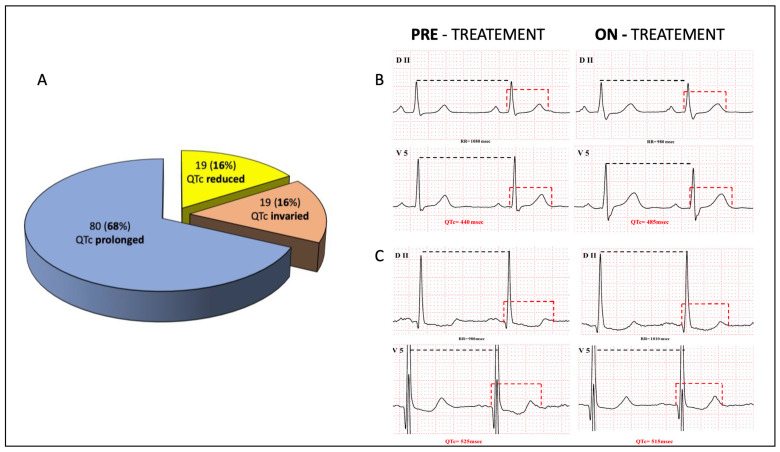
Spectrum of QTc interval changes during disopyramide treatment. In panel (**A**), the spectrum of QT variation following disopyramide therapy is reported. Panel (**B**) shows a case of QT prolongation during treatment, whereas Panel (**C**) represents a patient with QT shortening while on disopyramide.

Specifically, 19 (16%) patients showed reduced QTc from baseline (mean reduction 10 [8; 14] msec), 19 (16%) had no difference, while 80 (68%) patients had a prolonged QTc interval of 27 [19; 37] msec (Figure 2). Patients who presented with a QTc < 480 msec (88/118, 70%) at baseline had a more significant prolongation compared to those with an abnormal baseline QTc (24 [7; 35] vs. 5 [2; 11] msec, *p* < 0.01). A significantly higher proportion of patients (56, 48%) presented with a QTc > 480 msec after 12 months of treatment (Table 2).

Thirty-one (26%) patients experienced side effects, in particular 29 of the anticholinergic type. Such symptoms led to the reduction of treatment in 5 (4%) patients (Table 4).

At the end of the study period, a total of 67/118 (%) patients suspended the treatment. Specifically, 53 (79%) underwent SRT because of ineffective response, 10 (15%) because of anti-cholinergic collateral effects, and 4 (6%) for QTc prolongation above 550 msec.

## 4. Discussion

Despite being largely used since the 1980s for patients with obstructive HCM, the real-world use of disopyramide and the markers of treatment response are still unknown. This is of critical relevance, since the recent development of disease-specific therapies, such as myosin inhibitors, challenges clinicians to find the appropriate sub-group as well as the correct disease stage for each drug [12].

The present study shows that disopyramide was underused since the 1980s in a historical and large cohort of patients with obstructive HCM, being offered to up to one-third of the patients. Such finding reflects several possible limitations related to the use of this treatment. Despite being recommended by scientific societies [5,13], there is a chronic supply shortage of disopyramide in several European countries, being an old medication without important commercial interest. Moreover, a certain lack of confidence by physicians might exist, many of whom are reluctant to use class I anti-arrhythmic agents in structural heart disease and fear possible complications related to disopyramide-induced augmentation of atrioventricular conduction and increased ventricular rate.

In our cohort, the efficacy of disopyramide was present in a quarter of patients, almost exclusively observed in patients who were slightly symptomatic, with a functional class NYHA I or II. Interestingly, responders were younger, had a smaller left atrium, less severe LVOT gradient, and a higher LV EF. These findings are in line with the drug physiopathological effect, mainly driven by its negative inotropic effect, which is the main mechanistic driver of reduction in LVOT gradients; it is less potent in patients who demonstrate less HCM-induced hypercontractility [6,7]. Taken together, these finding suggest that clinicians should not be discouraged from trying disopyramide in patients who are symptomatic and have high LVOT gradients despite administering the maximum doses of other negative inotropic drugs. This may be especially true in those patients with high-normal LVEF and non-enlarged LA, factors that were associated with response to disopyramide therapy in the current study. Moreover, no difference was observed between the maximum drug dose and patients on the minimum effective posology, suggesting that effective reduced dose of the drug might be similarly effective despite limiting adverse effects, which are dose-dependent. Such findings are in accordance with previous smaller reports from Habib et al. [8]. On the contrary, our data highlight that severely symptomatic patients, with a NYHA class III/IV and a remodeled myocardium, would hardly respond to disopyramide. In this subset of patients, direct referral to SRT might probably be the best therapeutic option. At the end of the study period, almost 50% of patients were proposed to be treated with an invasive SRT and almost exclusively in the incomplete and non-responders in the NYHA III/IV group.

Concerning the safety profile, no major sustained arrhythmias occurred during the whole study period. This is in line with current literature that shows freedom from cardiovascular death including sudden cardiovascular death, and with a total mortality similar to the general population [6,7]. QTc prolongation was observed in 68% of patients, and QTc prolongation above 500 msec in up to 28%. This was mainly correlated with an increase in QRS length [14], and only in four cases led to drug discontinuation because of increase in QTc above 550 msec. Interestingly, 32% of patients did not show significant QTc related changes or reduced length of repolarization. This is in line with the in vitro observations from Coppini et al. [11], which showed the multichannel inhibitory effects and the membrane stabilizing actions of disopyramide.

Disopyramide was overall well tolerated, and side effects, mainly anti-cholinergic, led to treatment interruption in 8% of patients. As a limitation and possible bias of the study, investigators did not propose systematic pyridostigmine for patients with severe side effects.

In conclusion, while awaiting approval from the regulatory agencies for disease specific therapies such as myosin inhibitors, HCM experts are evaluating the positioning of each specific therapy in current management algorithms. Despite surgeons’ concerns [15], negative inotropic drugs and myosin inhibitors may shape the current practice. Furthermore, as we have recently learned from the case of tafamidis, a progression-slowing drug for transthyretin-related amyloidosis [16], pricing may represent a key issue influencing patients’ access to treatment [16], particularly in less-developed economies. Based on the EXPLORER-HCM trial results, symptomatic patients with oHCM not responding fully to (or not tolerating) β-blockers and disopyramide should be considered for mavacamten treatment [17], potentially proposing the drug as a life-long therapy. Therefore, sustainability should be a relevant aspect of all treatments addressing left ventricular obstruction. The present study shows that, in a specific subset of slightly symptomatic patients with oHCM, a relatively inexpensive drug such as disopyramide might still play an important role. This would be a relevant factor for global experts when specific treatment algorithms would be proposed in the Guidelines for patients with symptomatic LVOT. Lastly, further prospective studies would be needed to compare negative inotropic drugs with myosin inhibitors, specifically in the subset of obstructive HCM patients with atrial fibrillation, as mavacamten does not exert any classic antiarrhythmic effect.

## 5. Limitations

This study was limited by its size and retrospective nature. Accordingly, only the association of the factors studied with response to disopyramide treatment could be investigated. However, the associations demonstrated in this study are in line with our knowledge of disease mechanisms in HCM and the mode of action of disopyramide.

## 6. Conclusions

Disopyramide was underused in oHCM but effective in reducing symptoms and LVOTO gradients in patients with slightly symptomatic patient with less severe disease phenotype with a safe pro-arrhythmic profile. Further prospective studies would be needed to ascertain the role of negative inotropes in the treatment algorithm of patients with slightly symptomatic obstructive HCM.

## Figures and Tables

**Table 1:** Baseline characteristics of the cohort at disopyramide therapy initiation.

	Variable	Total Cohort (n = 118)	Responders (n = 28)	Incomplete Responders (n = 39)	Non-Responders (n = 51)
**Demographic**					
	Age (ys)	55 ± 16	48 ± 9 *	51 ± 11	58 ± 12
	Male sex (n, %)	63 (53%)	16 (57%)	19 (49%)	28 (55%)
**Medical History**					
	NYHA Class I–II (n, %)	64 (54 %)	24 (86%) *	24 (62%)	16 (31%)
	NYHA Class III (n, %)	53 (46 %)	3 (11%)	15 (38%)	35 (69%) *
	NYHA Class IV (n, %)	1 (1.5%)	1 (3%)	0	0
	Atrial fibrillation (n, %)	32 (27%)	6 (21%)	11 (28%)	15 (29%)
	Syncope (n, %)	27 (0.12 %)	1 (4%) *	12 (31%)	14 (27%)
	PM/ICD (n, %)	11 (9%)	1 (4%) *	3 (8%)	7 (14%)
	NSVT (n, %)	22 (19%)	3 (11%)	6 (15%)	13 (25%)
	Cardiac Arrest (n, %)	1 (0.8%)	0	1 (2.5%)	0
**Treatments**					
	Beta-blockers (n, %)	99 (84 %)	22 (79%)	35 (90%)	42 (81%)
	Calcium-Antagonist (n, %)	14 (8 %)	2 (8%)	4 (10%)	8 (16%)
	Amiodarone (n, %)	12 (10 %)	1 (4%)	4 (10%)	7 (14%)
**Echocardiogram**					
	SAM (n, %)	95 (80%)	22 (78%)	33 (85%)	40 (78%)
	Left Atrial diameter (mm)	45 ± 7	42 ± 5 *	44 ± 6	46 ± 7
	LV Maximal Wall Thickness (mm)	22 ± 5	21 ± 4	22 ± 5	22 ± 6
	Resting LVOTO (mmHg)	72 ± 36	69 ± 21	70 ± 18	73 ± 22
	30 mmHg < LVOTO < 50 mmHg (n, %)	42 (36%)	13 (46%)	10 (26%)	19 (37%)
	LVOTO > 50 mmHg (n, %)	76 (64%)	15 (54 %)	29 (74 %) *	32 (63%)
	Maximal LVOTO (mmHg)	88 ± 35	71 ± 12	91 ± 13	85 ± 21
	LV Ejection Fraction (%)	66 ± 7	69 ± 8	66 ± 6	63 ± 8 *

Abbreviations: PM: pacemaker; ICD: implantable cardioverter defibrillator; NSVT: non-sustained ventricular tachycardia; SAM: systolic anterior motion of the mitral valve; LV: left ventricle; LVOTO: left ventricular outflow tract obstruction. * = p < 0.05.

**Table 2:** Electrocardiographic changes on disopyramide treatment.

Variables	Pre-Treatment	On Treatment	*p* Values
**HR (bpm)**	60 ± 8	59 ± 79	0.45
**PR (msec)**	178 ± 22	183 ± 24	<0.01
**AVB I (n)**	10 (8%)	22 (17%)	<0.01
**QRS (msec)**	101 ± 22	109 ± 26	0.10
**New Onset Bundle Branch Block (n)**	14 (11%)	20 (16%)	0.62
**QTc_max_ (msec)**	423 ± 29	475 ± 41	0.67
**QTc ≥ 480 msec**	30 (24%)	56 (48%)	<0.01
**QTc ≥ 500 msec**	14 (11%)	36 (28%)	<0.01

*Abbreviations: HR: Heart Rate.*

**Table 3:** Multivariable Predictors of response to disopyramide therapy.

	Univariable Analysis			Multivariable Analysis		
Variable	HR	95% CI	*p*-Value	HR	95% CI	*p*-Value
Age (per 10 decrease)	1.4	[0.5–3.6]	0.03			
NYHA Class I-II (n)	5.1	[2.3–11.2]	<0.01	1.5	[1.1–4.5]	0.03
Left atrial diameter (per 2 mm decrease)	2.1	[0.9–7.8]	<0.01			
LV EF (per 5 increase)	4.2	[1.3–9.9]	<0.01	1.9	[0.9–6.4]	0.07

**Table 4:** Collateral effects of patients undergoing disopyramide treatment.

	Total Cohort (n = 118)	Treatment Reduction	Treatment Suspension
**Anticholinergic collateral** **effects**	29 (24%)	5 (4%)	10 (8%)
Xerostomia/Xerophthalmia	13 (11%)	4 (3%)	8 (7%)
Stypsis	10 (8%)	1 (0.8%)	1 (0.8%)
Blurred vision	2 (2%)	1 (0.8%)	0
Urinary Retention	4 (3%)	0	1
**Sustained Ventricular** **Arrhythmias**	0	-	-
**Torsade de Pointes**	0	-	-

## Data Availability

Data would be shared upon reasonable request.
